# The Global Otolaryngology–Head and Neck Surgery Workforce

**DOI:** 10.1001/jamaoto.2023.2339

**Published:** 2023-08-31

**Authors:** Beatriz Petrucci, Samuel Okerosi, Rolvix H. Patterson, Sara B. Hobday, Valerie Salano, Christopher J. Waterworth, Robert M. Brody, Holly Sprow, Blake C. Alkire, Johannes J. Fagan, Sharon Ovnat Tamir, Carolina Der, Mahmood F. Bhutta, Ivy W. Maina, Jonathan C. Pang, Davina Daudu, Allan G. Mukuzi, Tarika Srinivasan, Carolina A. Pietrobon, Sheng-Po Hao, Doreen Nakku, Amina Seguya, Taseer F. Din, Olga Djoutsop Mbougo, Lilian W. Mokoh, Farizeh Jashek-Ahmed, Tyler J. Law, Elizabeth A. Holt, Ali Haider Bangesh, Yilkal Zemene, Titus S. Ibekwe, Oumar Raphiou Diallo, Jaqueline Alvarado, Wakisa K. Mulwafu, John E. Fenton, Adrian M. Agius, Pavel Doležal, Édouard Amani Mudekereza, Karen M. Mojica, Ricardo Silva Rueda, Mary Jue Xu

**Affiliations:** 1 Unified Health System, Ministry of Health, Brazil; 2 Ear Nose and Throat Department, Kenyatta National Hospital, Nairobi, Kenya; 3 Department of Head and Neck Surgery & Communication Sciences, Duke University School of Medicine, Durham, North Carolina; 4 Department of Otorhinolaryngology-Head and Neck Surgery, Perelman School of Medicine, University of Pennsylvania, Philadelphia; 5 Ear Nose and Throat Department, Nyahururu County Hospital, Nyahururu, Kenya; 6 Nossal Institute for Global Health, Department of Audiology and Speech Pathology, University of Melbourne, Melbourne, Victoria, Australia; 7 Department of Otorhinolaryngology–Head & Neck Surgery, University of Pennsylvania, Philadelphia; 8 Tufts University School of Medicine, Boston, Massachusetts; 9 Department of Otolaryngology–Head and Neck Surgery, Massachusetts Eye and Ear Infirmary, Boston; 10 Division of Otolaryngology, University of Cape Town, Cape Town, South Africa; 11 Department of Otolaryngology/Head and Neck Surgery, Samson Assuta Ashdod University Hospital, Ben Gurion University of the Negev, Israel; 12 Universidad del Desarrollo, Facultad de Medicina, Clínica Alemana de Santiago, Hospital Dr Luis Calvo Mackenna, Chile; 13 Brighton & Sussex Medical School, Brighton, United Kingdom; 14 Department of Otolaryngology–Head and Neck Surgery, University of California, Irvine; 15 Faculty of Surgery, University of Western Australia, Perth, Western Australia, Australia; 16 Department of Otorhinolaryngology Head and Neck Surgery, University of Nairobi, Kenya; 17 Medical student, Harvard Medical School, Boston, Massachusetts; 18 Universidade do Vale do Taquari, Brazil; 19 Department of Otolaryngology Head and Neck Surgery, Shin Kong Wu Ho-Su Memorial Hospital, Fu-Jen University, Taiwan; 20 Department of Otolaryngology Head and Neck Surgery, Mbarara University of Science and Technology, Uganda; 21 Department of Otolaryngology Head and Neck Surgery, Mulago National Referral Hospital, Uganda; 22 Division of Pediatric Otolaryngology, Head-Neck Surgery, Department of Otolaryngology, Head-Neck Surgery, Stanford University, Stanford, California; 23 Bel Campus University of Technology, Kinshasa, Democratic Republic of Congo; 24 Kenyatta University Teaching Research and Referral Hospital, Nairobi, Kenya; 25 The International Center for Recurrent Head and Neck Cancer, the Royal Marsden Hospital, London, United Kingdom; 26 Department of Anesthesia & Perioperative Care, University of California, San Francisco; 27 The Eisdell Moore Centre for Hearing and Balance Research, The University of Auckland, Auckland, New Zealand; 28 STMU Shifa College of Medicine, Islamabad, Pakistan; 29 St Paul’s Hospital Millennium Medical College, Addis Ababa, Ethiopia; 30 University of Abuja and University of Abuja Teaching Hospital, Abuja, Nigeria; 31 University of Conakry Médical Faculty, Conakry, Guinea; 32 Pan-American Otolaryngology Association, Miami, Florida; 33 Department of Surgery, Kamuzu University of Health Sciences, Blantyre, Malawi; 34 Department of Otorhinlaryngology–Head and Neck Surgery, University of Limerick, Limerick, Ireland; 35 Department of Otorhinolaryngology, University of Malta, Malta; 36 Department of Otorhinolaryngology and Head and Neck Surgery, Slovak Medical University, Bratislava, Slovakia; 37 Hôpital Provincial Général de Référence de Bukavu, Université Catholique de Bukavu, Bukavu, Democratic Republic of the Congo; 38 Department of otolaryngology, Vivian Pellas Hospital, Managua, Nicaragua; 39 Servicio de Otorrinolaringología, Bogota, Hospital Militar Central, Bogata, Colombia; 40 Department of Otolaryngology–Head and Neck Surgery, University of California, San Francisco

## Abstract

**Importance:**

A core component of delivering care of head and neck diseases is an adequate workforce. The World Health Organization report, *Multi-Country Assessment of National Capacity to Provide Hearing Care*, captured primary workforce estimates from 68 member states in 2012, noting that response rates were a limitation and that updated more comprehensive data are needed.

**Objective:**

To establish comprehensive workforce metrics for global otolaryngology–head and neck surgery (OHNS) with updated data from more countries/territories.

**Design, Setting, and Participants:**

A cross-sectional electronic survey characterizing the OHNS workforce was disseminated from February 10 to June 22, 2022, to professional society leaders, medical licensing boards, public health officials, and practicing OHNS clinicians.

**Main Outcome:**

The OHNS workforce per capita, stratified by income and region.

**Results:**

Responses were collected from 121 of 195 countries/territories (62%). Survey responses specifically reported on OHNS workforce from 114 countries/territories representing 84% of the world’s population. The global OHNS clinician density was 2.19 (range, 0-61.7) OHNS clinicians per 100 000 population. The OHNS clinician density varied by World Bank income group with higher-income countries associated with a higher density of clinicians. Regionally, Europe had the highest clinician density (5.70 clinicians per 100 000 population) whereas Africa (0.18 clinicians per 100 000 population) and Southeast Asia (1.12 clinicians per 100 000 population) had the lowest. The OHNS clinicians deliver most of the surgical management of ear diseases and hearing care, rhinologic and sinus diseases, laryngeal disorders, and upper aerodigestive mucosal cancer globally.

**Conclusion and Relevance:**

This cross-sectional survey study provides a comprehensive assessment of the global OHNS workforce. These results can guide focused investment in training and policy development to address disparities in the availability of OHNS clinicians.

## Key Points

**Question:**

What is the number of otolaryngology–head and neck surgery (OHNS) care clinicians per capita worldwide?

**Findings:**

In this cross-sectional survey study including OHNS organizations and clinicians, respondents from 114 countries comprising 84% of the world population estimate a workforce density of 2.19 OHNS clinicians per 100 000 population. Variations were noted by World Health Organization regions and World Bank income groups.

**Meaning:**

This comprehensive assessment of OHNS workforce provides a data-driven approach to surgical capacity-building programs and policies worldwide.

## Teaser

This cross-sectional study assesses the worldwide number and density, as well as scope of practice and availability of training programs, of otolaryngology–head and neck surgery clinicians.

## Introduction

Five billion people worldwide lack access to safe, timely, and affordable surgical care, which is needed to address the estimated 30% of the global burden of disease that requires surgical intervention.^1,2,3^ The 2015 Lancet Commission on Global Surgery highlighted human resources as the “backbone of health-care delivery systems” and proposed 20 surgical, anesthetic, and obstetric clinicians per 100 000 population as a workforce target, noting poorer health outcomes with lower surgical, anesthetic, and obstetric clinician density.^2^ While this was not stratified by type of surgical clinician, the World Health Organization (WHO) has previously suggested a minimum threshold of 1 otolaryngology–head and neck surgery (OHNS) clinician per 100 000 population.^4^ Additionally, the WHO report *Global Strategy on Human Resources for Health: Workforce 2030* further notes that a sustainable health workforce is critical to achieving the sustainable development goals.^5^ Quantitatively monitoring the surgical workforce is necessary to drive focused capacity-building and innovation in care delivery.

Otolaryngology–head and neck surgery conditions represent a considerable and underprioritized burden of surgical disease that disproportionately affects low- and middle-income countries (LMICs). Head and neck cancers are the seventh most common cancer, with an estimated economic burden of $535 billion between 2018 and 2030.^6,7^ While 80% of the disability-adjusted life-years from cancer are lost in LMICs, only 5% of resources are invested in cancer care in these settings.^8^ Furthermore, hearing loss is the fourth leading cause of disability, with 80% of those with hearing loss residing in LMICs.^9^ Hearing loss is associated with long-term sequelae, such as reduced educational attainment, decreased employment rate, and an increased risk of dementia.^10,11,12^ To deliver high-quality care for OHNS conditions globally, and especially in resource-constrained health care systems, the workforce needs to be assessed as has been done for other surgical subspecialties.^13,14,15^

The most comprehensive OHNS workforce assessment with a uniform method was the WHO Multi-Country Assessment of National Capacity to Provide Hearing Care, which included workforce estimates from 68 member states in 2012.^16^ The study noted that clinician density varied with country income classification; for example, the African Region had the lowest proportion of countries with at least 1 clinician per million population and overlapped with some of the highest prevalence of hearing loss.^16^ More recent regional OHNS workforce studies have further highlighted disparities in the availability of OHNS clinicians.^17,18,19,20,21^ Given advances in regional capacity-building and surgical policies through development of National Surgical, Obstetric and Anesthesia Plans, there is a need for an updated assessment of the OHNS workforce. Therefore, the Global OHNS Initiative, an international research collaborative, aimed to characterize the global OHNS workforce density, presence of training programs, and scope of care provided by the OHNS workforce.

## Methods

### Study Design

This survey (Supplement 1) was designed to quantify the OHNS workforce, presence of training programs, and scope of OHNS care. The survey was disseminated through email. The survey was translated into the 6 official United Nations languages (English, Spanish, French, Chinese, Arabic, and Russian) and was designed to be completed in approximately 10 minutes. The study protocol was approved by the Research Ethics Board of the Hospital of the University of Pennsylvania. Participants provided informed consent; there was no financial compensation.

### Public Involvement

This survey was developed through a collaborative process with international authors and members of the Global OHNS Initiative, as well as experts in biostatistics and survey design (Supplement 1). The Global OHNS Initiative is an international research collaborative involving otolaryngologists, clinicians providing head and neck care, trainees, and students. The 271 members to date are from 58 countries with 51% of members from LMICs. Over the course of 2 years, the survey was designed through multiple monthly review meetings by members of the Global OHNS Initiative to intentionally collect international and diverse perspectives on the study aims, survey wording, and dissemination plan across countries/territories. In addition to these meetings, experts in biostatistics and survey design were consulted. Finally, dissemination of the survey was also supported through outreach by members of the Global OHNS Initiative. Those who participated in the study design, dissemination, data analysis, manuscript writing, and review are included as coauthors of this report. This study was open to input from members of the Global OHNS Initiative during organizational meetings and an open comment period. Those who provided input during this process are acknowledged herein as members of the Global OHNS Initiative.

### Clinician Definitions

International input was used to refine globally applicable definitions of clinician. An OHNS/ear nose and throat (ENT) physician was defined as a “doctor with a medical degree who has undergone specialized or accredited training in managing conditions of the ear, nose, and throat and head and neck. This does not include trainees.” Additional definitions are provided in eTable 1 in Supplement 2.

### Data Collection

All consent and survey responses were collected via email using the encrypted Research Electronic Data Capture (REDCap) database.^22^ Eligible participants included leaders of OHNS professional societies; leaders of medical licensing boards; leaders of public health government agencies, such as ministry of health departments; and OHNS professionals from the 194 WHO member states and Taiwan. Aligning with the method of similar workforce surveys,^23^ national and regional OHNS professional societies were first contacted, using professional networks and publicly available contact information. If there was no national professional society, then leadership of medical licensing boards, leadership of public health government agencies, and practicing clinicians were contacted. These participants were identified through professional networks or publicly available information from academic institutions and publications.

Workforce estimates were reported values by participants, from sources including data in databases, national reports, and professional networks. Email outreach began February 10, 2022, and was first performed through the REDCap platform. Respondents were given 2 to 3 weeks to respond, and 3 rounds of outreach were performed. Personal networks were used for subsequent rounds of email outreach. Additional contacts were located through online searches for medical licensing boards and clinician in countries/territories without formal professional organizations. Given that there were multiple rounds of outreach for each country/territory, there were multiple responses for some countries/territories. Data collection ended June 22, 2022, when few responses were obtained with additional rounds of email outreach.

### Statistical Analysis

Countries were categorized by WHO regions (African Region, Region of the Americas, Southeast Asia Region, European Region, Eastern Mediterranean Region, and Western Pacific Region) and World Bank income groups (low-, lower-middle, upper-middle, and high-income countries).^24,25^ Country population was obtained from World Bank estimates, except for Taiwan and Niue, which were obtained from the Center for Intelligence Agency World Factbook.^26,27^

For countries/territories with multiple workforce estimates, responses were prioritized in the following order: (1) professional society, (2) licensing board, (3) Ministry of Health, and (4) practicing clinicians. For any outliers for OHNS workforce estimates, the number was confirmed with the survey participant or member of the research team practicing in the same region. If there were multiple responses from the same country/territory at the same priority level for source of information, discrepancies in data were evaluated for congruity, and the average of the responses was used.

Data analysis was performed using Stata, version 15.1 (StataCorp LLC), aside from calculation of the Cliff δ value for effect size measurement, which was performed in R Project, version 4.1, for Statistical Computing (R Foundation for Statistical Computing), using the package rcompanion. Weighted averages were used to calculate density and characteristics of clinicians by WHO regions and World Bank income groups. The Cliff δ test was used to compare the proportion of OHNS clinicians caring for select disease processes (surgical management of ear and hearing care, surgical management of rhinologic and sinus diseases, surgical management of benign laryngeal disorders, surgical management of mucosal cancers, surgical management of thyroid disease, cleft lip and palate repair, and facial trauma) and World Bank income group.^28^ For this comparison, variables were dichotomized. Coverage by OHNS was dichotomized to greater than 50% and less than or equal to 50% OHNS coverage. Income groups were dichotomized to compare high-income countries with the remaining low- and middle-income countries. A positive value would indicate an association with more OHNS coverage in high-income countries compared with LMICs for the disease area. A negative Cliff δ effect size would indicate that more OHNS coverage existed in LMICs compared with high-income countries for the disease area.

## Results

### Survey Responses

**Figure 1.**
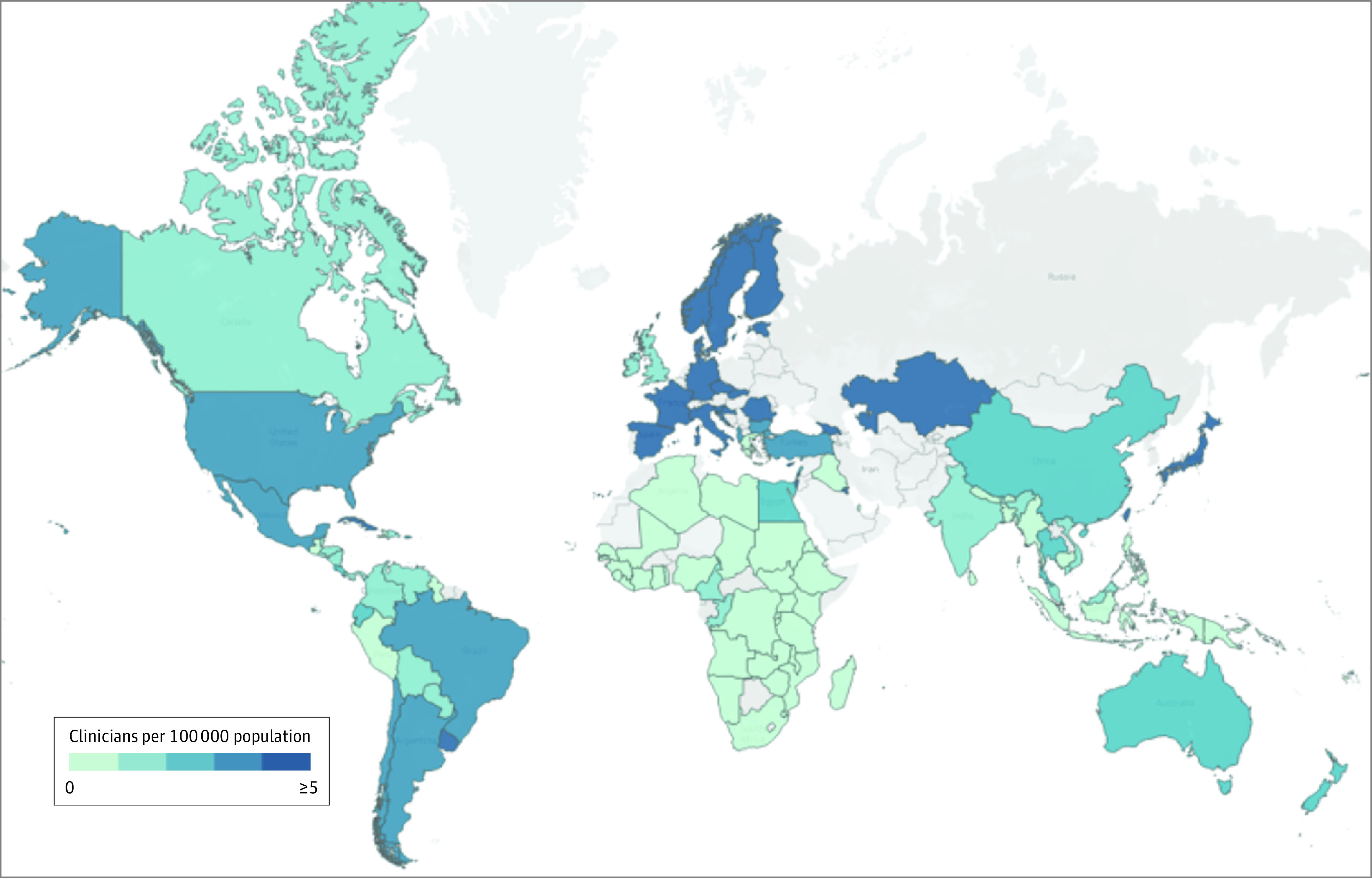
Otolaryngology–Head and Neck Surgery Ear, Nose, and Throat Clinicians Per Capita Countries/territories in gray are without estimates.

We collected responses from 121 of 195 countries/territories (62%). Of all responses, 114 countries specifically reported OHNS workforce density, representing 84% of the world’s population (Figure 1; eTable 2 in Supplement 2). Among the 114 OHNS workforce estimates used to assess OHNS density, 64% (n = 73) were from professional medical societies; 18% (n = 21) from government sources, such as ministries of health; 8% (n = 9) from medical licensing boards; and 10% (n = 11) from other sources. The most common additional source was personal estimates of the national workforce by practicing OHNS clinicians not otherwise associated with a professional society, licensing board, or government body.

Respondents from 8 countries (Iraq, Libya, Pakistan, Qatar, Senegal, Syria, Greece, and Saudi Arabia) did not provide workforce estimates. There were multiple responses from 16 countries (eTable 3 in Supplement 2). Survey responses varied by region, with the highest coverage in the Southeast Asian (11 of 13 countries [85%]) and African (34 of 47 countries [72%]) regions (eFigure 1 in Supplement 2).

### Workforce Density and Characteristics

**Figure 2.**
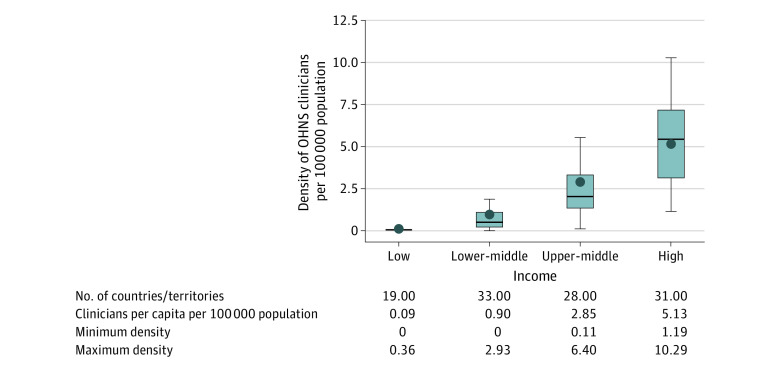
Otolaryngology–Head and Neck Surgery (OHNS) Workforce Per Capita by World Bank Income Group Range and quartiles represented by the box plot; outliers for the box plots are excluded. The horizontal line within the box represents the median value and the dot within the box represents the mean value. The top end of the box represents the 75th quartile and the bottom of the box represents the 25th quartile. The very top of the vertical line, indicated by the short horizontal bar, represents the maximum value. The very bottom of the vertical line, indicated by the short horizontal bar, represents the minimum value. Per capita by World Bank income group represented by the point in the box plot.

**Figure 3.**
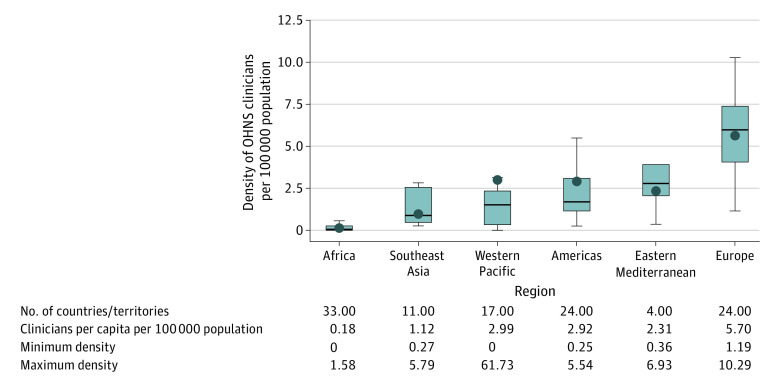
Otolaryngology–Head and Neck Surgery (OHNS) Workforce Per Capita by World Health Organization Region Range and quartiles represented by the box plot; outliers for the box plots are excluded. The horizontal line within the box represents the median value and the dot within the box represents the mean value. The top end of the box represents the 75th quartile and the bottom of the box represents the 25th quartile. The very top of the vertical line, indicated by the short horizontal bar, represents the maximum value. The very bottom of the vertical line, indicated by the short horizontal bar, represents the minimum value. Per capita by World Health Organization region represented by the point in the box plot. The regional per capita of the Western Pacific region is outside of the IQR given exclusion of extreme outliers for the visualization using the box plots to show dispersion of these data.

Overall, OHNS workforce density for the 114 countries/territories was 2.19 (range, 0-61.7) clinicians per 100 000 population. Clinician density was highest in high-income countries (Figure 2). Regionally, Europe had the highest clinician provider density (5.70 clinicians per 100 000 population) whereas Africa (0.18 clinicians per 100 000 population) and Southeast Asia (0.18 and 1.12 clinicians per 100 000 population) had the lowest. (Figure 3). Among all countries/territories, 42% (n = 48) had 1 or fewer clinicians per 100 000 population: this included 0% of high (0 of 31), 21% (6 of 28) of upper-middle, 70% (23 of 33) of lower-middle, and 100% (19 of 19) of low-income countries. By WHO regions, 91% (30 of 33) of African, 55% (6 of 11) of Southeast Asian, 41% (7 of 17) of Western Pacific, 25% (1 of 4) Eastern Mediterranean, and 17% (4 of 24) of American countries had a density of clinicians equal to or less than 1 per 100 000 population.

The median percentage of female OHNS clinicians per country/territory was 40.0% (IQR, 30.0%-42.0%). The lowest proportions of female clinicians were reported in the Eastern Mediterranean (n = 3; median, 26.0%) and Southeast Asia regions (n = 10; median, 15.0%) (eTable 4 in Supplement 2). The median percentage of clinicians per country/territory based in urban practices at tertiary medical centers was 50.0% (IQR, 46.0%-65.0%), with a greater proportion in low-income countries (median, 90.0%) compared with high- and middle-income countries (median high-income, 50.0%; upper-middle, 50.0%; and lower-middle, 30.0%). The median percentage of clinicians per country/territory who practiced either part time or full time in the public sector as opposed to the private sector was 70.0% (IQR, 30.0%-80.0%) and the median percentage of clinicians per country/territory who trained outside of their current country of practice was 5.0% (IQR, 5.0%-30.0%).

### Scope of Practice

**Figure 4.**
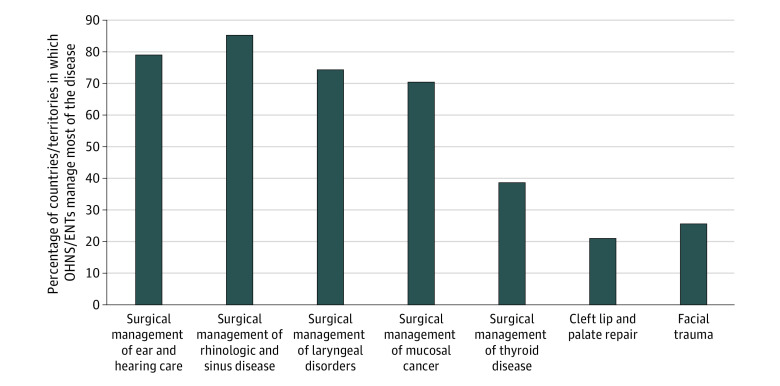
Percentage of Countries/Territories in Which Otolaryngology–Head and Neck Surgeons (OHNS) Manage Most of the Disease Processes ENT indicates ear, nose, and throat.

More than 70% of countries/territories reported that the OHNS workforce provided at least 50% of surgical care for ear and hearing care conditions, rhinologic and sinus conditions, benign laryngeal disorders, and mucosal cancers (Figure 4). Other surgical subspecialties, such as general surgery, plastic surgery, and oral and maxillofacial surgery, were reported to provide a substantial portion of surgical care for thyroid disease, cleft lip and palate, and facial trauma (eFigure 2 in Supplement 2). Higher proportions of surgical ear and hearing care, rhinology and sinus, benign laryngeal, and upper aerodigestive cancers are managed by OHNS clinicians in high-income countries (eTable 5 in Supplement 2).

### Training Programs

Among 115 countries/territories, 23 (20%) reported the absence of any OHNS training programs. The Western Pacific and African Regions had the lowest proportion of countries with OHNS training programs (Table).

**Table.** Proportion of Countries/Territories Without OHNS/ENT Training Programs

Variable	No.	Countries without training program, No. (%)
Total	115	23 (20)
World Bank income groups		
High	32	2 (6)
Upper middle	29	8 (28)
Lower middle	33	7 (21)
Low	18	5 (28)
Total	112	22 (20)
World Health Organization regions		
Americas	24	3 (13)
Africa	33	10 (30)
Eastern Mediterranean^a^	9	0
Europe	24	2 (8)
Southeast Asia	11	1 (9)
Western Pacific	17	7 (41)
Total	114	23 (20)

Abbreviations: ENT, ear, nose, and throat; OHNS, otolaryngology–head and neck surgery.

^a^ Workforce estimates were available for 4 countries.

## Discussion

To our knowledge, this study presents the most comprehensive assessment of the OHNS workforce to date, with estimates representing 84% of the world’s population. There are 2.19 OHNS clinicians per 100 000 population globally, with the distribution of this workforce varying by income group and region. There is a particular need for capacity-building in lower-income countries and in the African and Southeast Asian WHO regions. The LMICs had lower OHNS workforce densities (0.09 clinicians per 100 000 people in low-income countries, 0.90 in lower-middle income countries, and 2.85 in upper-middle income countries) than high-income countries (5.13 clinicians per 100 000 people), despite having 85% of the global population with OHNS conditions, such as head and neck cancer and hearing loss.^7,9^

Our results align with an OHNS workforce analysis conducted by Kamenov et al,^18^ which similarly reported that 78% of low-income countries had fewer than 0.1 OHNS clinicians per 100 000 and 88% of LMICs had fewer than 1 OHNS clinicians per 100 000. As Kamenov et al incorporated several sources of data spanning a 10-year period, our analysis provides an updated understanding of the global OHNS workforce with additional insights into the scope of practice, practice setting, and presence of training programs. Additionally, this study provides an updated estimate from more countries than the WHO *Multi-Country Assessment of National Capacity to Provide Hearing Care*, which collected workforce estimates from 68 member states in 2012.^16^

Results from this study are furthermore in line with regional reports. In a workforce assessment of sub-Saharan Africa, Mulwafu et al^19^ reported a density of 0.12 OHNS clinicians per 100 000 population for sub-Saharan Africa, similar to the density of 0.18 clinicians per 100 000 population presented in this study. These studies both displayed stark intercountry differences in the OHNS workforce; Mulwafu et al reported 246 OHNS clinicians in South Africa and 2 OHNS providers in Malawi, which aligns with our findings ranging from 0 to 300 clinicians per country in sub-Saharan Africa. Our study’s workforce estimates in Europe were also similar to a survey by Verkerk et al^20^ of the OHNS workforce in Eastern Europe, ranging from 3.6 to 12.3 clinicians per 100 000 population. Additionally, Latin America workforce estimates by Stolovitzky et al^21^ reported estimates ranging from 0.4 to 2.7 OHNS clinicians per 100 000 population, which was similar to the range of this study. In comparison with other subspecialties, the global OHNS workforce density of 2.19 clinicians per 100 000 population was greater than neurosurgery (0.36 clinicians per 100 000) and less than anesthesia (6.09 clinicians per 100 000).^23,29^

To create an expanded and sustained workforce, urgent investment is needed to increase regional general and subspecialty OHNS training programs alongside innovative task-sharing models. These task-sharing models include strategies to train general practitioners, nurses, community health workers, and others to provide elements of care for conditions of the head and neck.^17^ Collaborations such as among the College of Surgeons of East, Central and Southern Africa and the Pacific Island countries/territories have created regional educational programs by leveraging resources from several institutions or countries to build comprehensive training models.^30,31^ Investment in these capacity-building models will be critical to foster a desperately needed workforce. It is noteworthy that Mulwafu et al^19^ reported that between 2009 and 2015, OHNS workforce density decreased in some sub-Saharan African countries due to population growth and limited OHNS training programs. Because training of OHNS clinicians and subspecialists takes many years,^32^ task sharing may help address the current workforce shortfall.^18,33^ Finally, workforce capacity-building must be accompanied by financing to support the infrastructure, equipment, and multidisciplinary services needed to provide care. This includes efforts to train audiologists, speech and language therapists, oncologists, and other multidisciplinary team members required to provide comprehensive OHNS care.

### Limitations

This study had several limitations. First, its validity depends on the reporting accuracy of key stakeholders in each country relying on variable data sources (eg, society records, licensing board records, ministry of health records, or personal knowledge). This survey may be subject to response bias for several reasons: OHNS density could be overestimated to improve public perception or underestimated to increase funding opportunities for training programs. However, the regional OHNS workforce estimates presented herein were within the same order of magnitude of data presented in previous publications. Second, our study may underestimate the number of OHNS clinicians where nonphysician personnel provide such care, such as clinical officers or nurse practitioners/nurse specialists. Additionally, national workforce estimates do not illustrate intracountry variation in density, such as differences between urban and rural regions.^34^ Finally, this study sought 1 response per country/territory and a survey response rate was not recorded; future studies ought to consider collecting survey response rate.

Future research should develop detailed, country-specific workforce characterizations that can be used to guide capacity-building efforts, such as strengthening OHNS training through training program development and the creation of pertinent health policy. Additional studies may also determine the subspecialty OHNS workforce and access to subspecialty training, although it must be recognized that subspecialization may not be possible in countries with a limited workforce. These data also ought to be assessed with other metrics, including health outcomes, to establish benchmarks for the minimum OHNS clinician density. In addition, to guide resource allocation for workforce expansion, more work is needed to characterize the accessibility and quality of OHNS training programs. Most importantly, we hope these results can be leveraged to develop and expand capacity-building efforts for head and neck care providers.

## Conclusions

To our knowledge, this Global OHNS Initiative workforce cross-sectional survey is the most comprehensive study of the OHNS global workforce, highlighting disparities in OHNS clinician, density, regional needs for capacity-building, and opportunities for strengthening OHNS training. Furthermore, this study emphasizes the critical need for OHNS workforce, especially for ear and hearing care, rhinologic conditions, and upper mucosal aerodigestive diseases. Using these results, the global OHNS workforce can be strengthened to improve access to essential OHNS care around the world.

## Notes

Supplement 1. **eMethods.** Workforce Survey

Supplement 2. **eFigure 1.** Survey Responses by World Health Organization Region **eFigure 2.** Disease Management by Specialties Other Than Otolaryngology–Head and Neck Surgery **eTable 1.** Definitions of Care Providers **eTable 2.** Number of Otolaryngology–Head and Neck Surgery Providers and Provider Density by Country **eTable 3.** Duplicate Workforce Estimates and Sources **eTable 4.** Provider or Practice Characteristics **eTable 5.** Disease Coverage by Income Group

Supplement 3.
Data Sharing Statement
