# Supplementary material for: The Global Otolaryngology–Head and Neck Surgery Workforce
Source: JAMA Otolaryngol Head Neck Surg. 2023 Aug 31;149(10):904–11. doi: 10.1001/jamaoto.2023.2339 (PMC10472262; doi:10.1001/jamaoto.2023.2339)
Supplement: Supplement 1. — eMethods. Workforce Survey [file jamaotolaryngolheadnecksurg-e232339-s001.pdf]

Workforce Survey

This study asks questions about the otolaryngology-head and neck surgery (OHNS)/ ear, nose and throat (ENT) workforce in your country. We are asking you to take part in this survey because you are either a) a leader in health care within your country or b) a practitioner who may know the workforce estimates. More accurate and comprehensive workforce estimates are needed to direct future research, policy and programmatic interventions.

The survey is approximately 10 minutes to complete. You can withdraw participation at any point. If you are unable to take part in the study, let our team know and we will reach out to others.

For questions, please contact the the Global Otolaryngology-Head and Neck Initiative (globalohns.workforcesurvey@gmail.com) or the Principal Investigator, Blake Alkire (balkire@partners.org) . For questions about your rights as a research subject or any concerns directed towards someone not involved in this research, please contact the Partners Human Research Committee at +1 (857) 282-1900.

Please provide your signature. ______________________

Respondent Information

Select the country where you work/ currently live:

◯ Afghanistan
◯ Albania
◯ Algeria
◯ Andorra
◯ Angola
◯ Antigua and Barbuda
◯ Argentina
◯ Armenia
◯ Australia
◯ Austria
◯ Azerbaijan
◯ Bahamas
◯ Bahrain
◯ Bangladesh
◯ Barbados
◯ Belarus
◯ Belgium
◯ Belize
◯ Benin
◯ Bhutan
◯ Bolivia
◯ Bosnia and Herzegovina
◯ Botswana
◯ Brazil
◯ Brunei Darussalam
◯ Bulgaria
◯ Burkina Faso
◯ Burundi
◯ Cambodia
◯ Cameroon
◯ Canada
◯ Cape Verde
◯ Central African Republic
◯ Chad
◯ Chile
◯ China
◯ Colombia
◯ Comoros
◯ Congo (Republic of the Congo)
◯ Congo (Democratic Republic of the Congo)
◯ Cook Islands
◯ Costa Rica
◯ Côte d'Ivoire
◯ Croatia
◯ Cuba
◯ Cyprus
◯ Czech Republic
◯ Denmark
◯ Djiboutia
◯ Dominica
◯ Dominican Republic
◯ Ecuador
◯ Egypt
◯ El Salvador
◯ Equatorial Guinea
◯ Eritrea
◯ Estonia
◯ Ethiopia
◯ Fiji
◯ Finland
◯ France
◯ Gabon
◯ Gambia
◯ Georgia
◯ Germany
◯ Ghana
◯ Greece
◯ Grenada
◯ Guatemala

◯ Guinea
◯ Guinea-Bissau
◯ Guyana
◯ Haiti
◯ Honduras
◯ Hungary
◯ Iceland
◯ India
◯ Indonesia
◯ Iran
◯ Iraq
◯ Ireland
◯ Israel
◯ Italy
◯ Jamaica
◯ Japan
◯ Jordan
◯ Kazakhstan
◯ Kenya
◯ Kiribati
◯ Korea (North, Democratic People's Republic of Korea)
◯ Korea (South, Republic of Korea)
◯ Kosovo
◯ Kuwait
◯ Kyrgyzstan
◯ Laos
◯ Latvia
◯ Lebanon
◯ Lesotho
◯ Liberia
◯ Libya
◯ Liechtenstein
◯ Lithuania
◯ Luxembourg
◯ Macedonia (the former Yugoslav Republic of)
◯ Madagascar
◯ Malawi
◯ Malaysia
◯ Maldives
◯ Mali
◯ Malta
◯ Marshall Islands
◯ Mauritania
◯ Mauritius
◯ Mexico
◯ Micronesia (Federated States of)
◯ Moldova (Republic of)
◯ Monaco
◯ Mongolia
◯ Montenegro
◯ Morocco
◯ Mozambique
◯ Myanmar (Burma)
◯ Namibia
◯ Nauru
◯ Nepal
◯ Netherlands
◯ New Zealand
◯ Nicaragua
◯ Niger
◯ Nigeria
◯ Niue
◯ Norway
◯ Oman
◯ Pakistan
◯ Palau
◯ Panama
◯ Papua New Guinea
◯ Paraguay
◯ Peru

◯ Philippines
◯ Poland
◯ Portugal
◯ Qatar
◯ Romania
◯ Russia
◯ Rwanda
◯ Saint Kitts and Nevis
◯ Saint Lucia
◯ Saint Vincent and the Grenadines
◯ Samoa
◯ San Marino
◯ Sao Tome and Principe
◯ Saudia Arabia
◯ Senegal
◯ Serbia
◯ Seychelles
◯ Sierra Leone
◯ Singapore
◯ Slovakia
◯ Slovenia
◯ Solomon Islands
◯ Somalia
◯ South Africa
◯ South Sudan
◯ Spain
◯ Sri Lanka
◯ Sudan
◯ Suriname
◯ Swaziland
◯ Sweden
◯ Switzerland
◯ Syrian Arab Republic (Syria)
◯ Taiwan
◯ Tajikistan
◯ Tanzania
◯ Thailand
◯ Timor-Leste
◯ Togo
◯ Tonga
◯ Trinidad and Tobago
◯ Tunisia
◯ Turkey
◯ Turkmenistan
◯ Tuvalu
◯ Uganda
◯ Ukraine
◯ United Arab Emirates
◯ United Kingdom
◯ United States of America
◯ Uruguay
◯ Uzbekistan
◯ Vanuatu
◯ Vatican City
◯ Venezuela
◯ Vietnam
◯ Yemen
◯ Zambia
◯ Zimbabwe

What describes your role? Check all that apply.	☐ A senior member of a professional otolaryngology/ ear nose and throat (ENT) society<br>☐ A senior member of a medical licensing board<br>☐ A senior member of a Ministry of Health<br>☐ A practicing otolaryngology-head and neck surgeon/ ear nose and throat (ENT) physician (does not include trainees)<br>☐ Other
What is your role if not listed above?	__________________________________

What is your country of origin?

◯ Afghanistan
◯ Albania
◯ Algeria
◯ Andorra
◯ Angola
◯ Antigua and Barbuda
◯ Argentina
◯ Armenia
◯ Australia
◯ Austria
◯ Azerbaijan
◯ Bahamas
◯ Bahrain
◯ Bangladesh
◯ Barbados
◯ Belarus
◯ Belgium
◯ Belize
◯ Benin
◯ Bhutan
◯ Bolivia
◯ Bosnia and Herzegovina
◯ Botswana
◯ Brazil
◯ Brunei Darussalam
◯ Bulgaria
◯ Burkina Faso
◯ Burundi
◯ Cambodia
◯ Cameroon
◯ Canada
◯ Cape Verde
◯ Central African Republic
◯ Chad
◯ Chile
◯ China
◯ Colombia
◯ Comoros
◯ Congo (Republic of the Congo)
◯ Congo (Democratic Republic of the Congo)
◯ Cook Islands
◯ Costa Rica
◯ Côte d'Ivoire
◯ Croatia
◯ Cuba
◯ Cyprus
◯ Czech Republic
◯ Denmark
◯ Djiboutia
◯ Dominica
◯ Dominican Republic
◯ Ecuador
◯ Egypt
◯ El Salvador
◯ Equatorial Guinea
◯ Eritrea
◯ Estonia
◯ Ethiopia
◯ Fiji
◯ Finland
◯ France
◯ Gabon
◯ Gambia
◯ Georgia
◯ Germany
◯ Ghana
◯ Greece
◯ Grenada
◯ Guatemala

◯ Guinea
◯ Guinea-Bissau
◯ Guyana
◯ Haiti
◯ Honduras
◯ Hungary
◯ Iceland
◯ India
◯ Indonesia
◯ Iran
◯ Iraq
◯ Ireland
◯ Israel
◯ Italy
◯ Jamaica
◯ Japan
◯ Jordan
◯ Kazakhstan
◯ Kenya
◯ Kiribati
◯ Korea (North, Democratic People's Republic of Korea)
◯ Korea (South, Republic of Korea)
◯ Kosovo
◯ Kuwait
◯ Kyrgyzstan
◯ Laos
◯ Latvia
◯ Lebanon
◯ Lesotho
◯ Liberia
◯ Libya
◯ Liechtenstein
◯ Lithuania
◯ Luxembourg
◯ Macedonia (the former Yugoslav Republic of)
◯ Madagascar
◯ Malawi
◯ Malaysia
◯ Maldives
◯ Mali
◯ Malta
◯ Marshall Islands
◯ Mauritania
◯ Mauritius
◯ Mexico
◯ Micronesia (Federated States of)
◯ Moldova (Republic of)
◯ Monaco
◯ Mongolia
◯ Montenegro
◯ Morocco
◯ Mozambique
◯ Myanmar (Burma)
◯ Namibia
◯ Nauru
◯ Nepal
◯ Netherlands
◯ New Zealand
◯ Nicaragua
◯ Niger
◯ Nigeria
◯ Niue
◯ Norway
◯ Oman
◯ Pakistan
◯ Palau
◯ Panama
◯ Papua New Guinea
◯ Paraguay
◯ Peru

◯ Philippines
◯ Poland
◯ Portugal
◯ Qatar
◯ Romania
◯ Russia
◯ Rwanda
◯ Saint Kitts and Nevis
◯ Saint Lucia
◯ Saint Vincent and the Grenadines
◯ Samoa
◯ San Marino
◯ Sao Tome and Principe
◯ Saudia Arabia
◯ Senegal
◯ Serbia
◯ Seychelles
◯ Sierra Leone
◯ Singapore
◯ Slovakia
◯ Slovenia
◯ Solomon Islands
◯ Somalia
◯ South Africa
◯ South Sudan
◯ Spain
◯ Sri Lanka
◯ Sudan
◯ Suriname
◯ Swaziland
◯ Sweden
◯ Switzerland
◯ Syrian Arab Republic (Syria)
◯ Taiwan
◯ Tajikistan
◯ Tanzania
◯ Thailand
◯ Timor-Leste
◯ Togo
◯ Tonga
◯ Trinidad and Tobago
◯ Tunisia
◯ Turkey
◯ Turkmenistan
◯ Tuvalu
◯ Uganda
◯ Ukraine
◯ United Arab Emirates
◯ United Kingdom
◯ United States of America
◯ Uruguay
◯ Uzbekistan
◯ Vanuatu
◯ Vatican City
◯ Venezuela
◯ Vietnam
◯ Yemen
◯ Zambia
◯ Zimbabwe

What is the country you performed the majority of your training in?

- ◯ Afghanistan
- ◯ Albania
- ◯ Algeria
- ◯ Andorra
- ◯ Angola
- ◯ Antigua and Barbuda
- ◯ Argentina
- ◯ Armenia
- ◯ Australia
- ◯ Austria
- ◯ Azerbaijan
- ◯ Bahamas
- ◯ Bahrain
- ◯ Bangladesh
- ◯ Barbados
- ◯ Belarus
- ◯ Belgium
- ◯ Belize
- ◯ Benin
- ◯ Bhutan
- ◯ Bolivia
- ◯ Bosnia and Herzegovina
- ◯ Botswana
- ◯ Brazil
- ◯ Brunei Darussalam
- ◯ Bulgaria
- ◯ Burkina Faso
- ◯ Burundi
- ◯ Cambodia
- ◯ Cameroon
- ◯ Canada
- ◯ Cape Verde
- ◯ Central African Republic
- ◯ Chad
- ◯ Chile
- ◯ China
- ◯ Colombia
- ◯ Comoros
- ◯ Congo (Republic of the Congo)
- ◯ Congo (Democratic Republic of the Congo)
- ◯ Cook Islands
- ◯ Costa Rica
- ◯ Côte d'Ivoire
- ◯ Croatia
- ◯ Cuba
- ◯ Cyprus
- ◯ Czech Republic
- ◯ Denmark
- ◯ Djiboutia
- ◯ Dominica
- ◯ Dominican Republic
- ◯ Ecuador
- ◯ Egypt
- ◯ El Salvador
- ◯ Equatorial Guinea
- ◯ Eritrea
- ◯ Estonia
- ◯ Ethiopia
- ◯ Fiji
- ◯ Finland
- ◯ France
- ◯ Gabon
- ◯ Gambia
- ◯ Georgia
- ◯ Germany
- ◯ Ghana
- ◯ Greece
- ◯ Grenada
- ◯ Guatemala

◯ Guinea
◯ Guinea-Bissau
◯ Guyana
◯ Haiti
◯ Honduras
◯ Hungary
◯ Iceland
◯ India
◯ Indonesia
◯ Iran
◯ Iraq
◯ Ireland
◯ Israel
◯ Italy
◯ Jamaica
◯ Japan
◯ Jordan
◯ Kazakhstan
◯ Kenya
◯ Kiribati
◯ Korea (North, Democratic People's Republic of Korea)
◯ Korea (South, Republic of Korea)
◯ Kosovo
◯ Kuwait
◯ Kyrgyzstan
◯ Laos
◯ Latvia
◯ Lebanon
◯ Lesotho
◯ Liberia
◯ Libya
◯ Liechtenstein
◯ Lithuania
◯ Luxembourg
◯ Macedonia (the former Yugoslav Republic of)
◯ Madagascar
◯ Malawi
◯ Malaysia
◯ Maldives
◯ Mali
◯ Malta
◯ Marshall Islands
◯ Mauritania
◯ Mauritius
◯ Mexico
◯ Micronesia (Federated States of)
◯ Moldova (Republic of)
◯ Monaco
◯ Mongolia
◯ Montenegro
◯ Morocco
◯ Mozambique
◯ Myanmar (Burma)
◯ Namibia
◯ Nauru
◯ Nepal
◯ Netherlands
◯ New Zealand
◯ Nicaragua
◯ Niger
◯ Nigeria
◯ Niue
◯ Norway
◯ Oman
◯ Pakistan
◯ Palau
◯ Panama
◯ Papua New Guinea
◯ Paraguay
◯ Peru

◯ Philippines
◯ Poland
◯ Portugal
◯ Qatar
◯ Romania
◯ Russia
◯ Rwanda
◯ Saint Kitts and Nevis
◯ Saint Lucia
◯ Saint Vincent and the Grenadines
◯ Samoa
◯ San Marino
◯ Sao Tome and Principe
◯ Saudia Arabia
◯ Senegal
◯ Serbia
◯ Seychelles
◯ Sierra Leone
◯ Singapore
◯ Slovakia
◯ Slovenia
◯ Solomon Islands
◯ Somalia
◯ South Africa
◯ South Sudan
◯ Spain
◯ Sri Lanka
◯ Sudan
◯ Suriname
◯ Swaziland
◯ Sweden
◯ Switzerland
◯ Syrian Arab Republic (Syria)
◯ Taiwan
◯ Tajikistan
◯ Tanzania
◯ Thailand
◯ Timor-Leste
◯ Togo
◯ Tonga
◯ Trinidad and Tobago
◯ Tunisia
◯ Turkey
◯ Turkmenistan
◯ Tuvalu
◯ Uganda
◯ Ukraine
◯ United Arab Emirates
◯ United Kingdom
◯ United States of America
◯ Uruguay
◯ Uzbekistan
◯ Vanuatu
◯ Vatican City
◯ Venezuela
◯ Vietnam
◯ Yemen
◯ Zambia
◯ Zimbabwe

In which country do you currently practice as a clinician?

- ◯ Afghanistan
- ◯ Albania
- ◯ Algeria
- ◯ Andorra
- ◯ Angola
- ◯ Antigua and Barbuda
- ◯ Argentina
- ◯ Armenia
- ◯ Australia
- ◯ Austria
- ◯ Azerbaijan
- ◯ Bahamas
- ◯ Bahrain
- ◯ Bangladesh
- ◯ Barbados
- ◯ Belarus
- ◯ Belgium
- ◯ Belize
- ◯ Benin
- ◯ Bhutan
- ◯ Bolivia
- ◯ Bosnia and Herzegovina
- ◯ Botswana
- ◯ Brazil
- ◯ Brunei Darussalam
- ◯ Bulgaria
- ◯ Burkina Faso
- ◯ Burundi
- ◯ Cambodia
- ◯ Cameroon
- ◯ Canada
- ◯ Cape Verde
- ◯ Central African Republic
- ◯ Chad
- ◯ Chile
- ◯ China
- ◯ Colombia
- ◯ Comoros
- ◯ Congo (Republic of the Congo)
- ◯ Congo (Democratic Republic of the Congo)
- ◯ Cook Islands
- ◯ Costa Rica
- ◯ Côte d'Ivoire
- ◯ Croatia
- ◯ Cuba
- ◯ Cyprus
- ◯ Czech Republic
- ◯ Denmark
- ◯ Djiboutia
- ◯ Dominica
- ◯ Dominican Republic
- ◯ Ecuador
- ◯ Egypt
- ◯ El Salvador
- ◯ Equatorial Guinea
- ◯ Eritrea
- ◯ Estonia
- ◯ Ethiopia
- ◯ Fiji
- ◯ Finland
- ◯ France
- ◯ Gabon
- ◯ Gambia
- ◯ Georgia
- ◯ Germany
- ◯ Ghana
- ◯ Greece
- ◯ Grenada
- ◯ Guatemala

◯ Guinea
◯ Guinea-Bissau
◯ Guyana
◯ Haiti
◯ Honduras
◯ Hungary
◯ Iceland
◯ India
◯ Indonesia
◯ Iran
◯ Iraq
◯ Ireland
◯ Israel
◯ Italy
◯ Jamaica
◯ Japan
◯ Jordan
◯ Kazakhstan
◯ Kenya
◯ Kiribati
◯ Korea (North, Democratic People's Republic of Korea)
◯ Korea (South, Republic of Korea)
◯ Kosovo
◯ Kuwait
◯ Kyrgyzstan
◯ Laos
◯ Latvia
◯ Lebanon
◯ Lesotho
◯ Liberia
◯ Libya
◯ Liechtenstein
◯ Lithuania
◯ Luxembourg
◯ Macedonia (the former Yugoslav Republic of)
◯ Madagascar
◯ Malawi
◯ Malaysia
◯ Maldives
◯ Mali
◯ Malta
◯ Marshall Islands
◯ Mauritania
◯ Mauritius
◯ Mexico
◯ Micronesia (Federated States of)
◯ Moldova (Republic of)
◯ Monaco
◯ Mongolia
◯ Montenegro
◯ Morocco
◯ Mozambique
◯ Myanmar (Burma)
◯ Namibia
◯ Nauru
◯ Nepal
◯ Netherlands
◯ New Zealand
◯ Nicaragua
◯ Niger
◯ Nigeria
◯ Niue
◯ Norway
◯ Oman
◯ Pakistan
◯ Palau
◯ Panama
◯ Papua New Guinea
◯ Paraguay
◯ Peru

◯ Philippines
◯ Poland
◯ Portugal
◯ Qatar
◯ Romania
◯ Russia
◯ Rwanda
◯ Saint Kitts and Nevis
◯ Saint Lucia
◯ Saint Vincent and the Grenadines
◯ Samoa
◯ San Marino
◯ Sao Tome and Principe
◯ Saudia Arabia
◯ Senegal
◯ Serbia
◯ Seychelles
◯ Sierra Leone
◯ Singapore
◯ Slovakia
◯ Slovenia
◯ Solomon Islands
◯ Somalia
◯ South Africa
◯ South Sudan
◯ Spain
◯ Sri Lanka
◯ Sudan
◯ Suriname
◯ Swaziland
◯ Sweden
◯ Switzerland
◯ Syrian Arab Republic (Syria)
◯ Taiwan
◯ Tajikistan
◯ Tanzania
◯ Thailand
◯ Timor-Leste
◯ Togo
◯ Tonga
◯ Trinidad and Tobago
◯ Tunisia
◯ Turkey
◯ Turkmenistan
◯ Tuvalu
◯ Uganda
◯ Ukraine
◯ United Arab Emirates
◯ United Kingdom
◯ United States of America
◯ Uruguay
◯ Uzbekistan
◯ Vanuatu
◯ Vatican City
◯ Venezuela
◯ Vietnam
◯ Yemen
◯ Zambia
◯ Zimbabwe

How many years have you been in practice?

____

How would you describe your practice setting? You can check multiple.	☐ Urban<br>☐ Rural<br>☐ Private<br>☐ Public
What is your age?	______________________________
What is your gender?	◯ Male<br>◯ Female<br>◯ Gender non-binary<br>◯ Different term ___________<br>◯ Prefer not to answer
How would you describe your gender?	______________________________

Workforce Roles

The following definitions are provided below for completion of this survey:

Otolaryngology-head and neck surgery (OHNS)/ear nose and throat (ENT) physician: Doctor with a medical degree who has undergone specialized or accredited training in managing conditions of the ear, nose, and throat and head and neck. This does not include trainees.

Audiologist service providers: Allied health professional who has received specialized training in hearing assessment. This includes both audiologists and audiology technicians.

Speech language pathologists/speech therapists: Allied health professionals specializing in management of voice and swallowing.

Otolaryngology clinical officers/ physician assistants: Allied health professionals who support care of patients with head and neck conditions. Practices under the supervision of an ear, nose, and throat physician.

Otolaryngology-specific nursing: Nurses who have developed specialized skills in ear, nose, and throat care and/or spend the majority of their time with patients with head and neck conditions.

Primary healthcare professionals with specific otolaryngology training: Primary care professionals who have undergone additional ear, nose, and throat training, which can include short courses.

Are the following health care professionals available where you work in your country?

	Yes	No	Unknown
Otolaryngology/ ear nose and throat physician	◯	◯	◯
Audiologist service providers	◯	◯	◯
Speech language pathologists/speech therapists	◯	◯	◯
Otolaryngology clinical officers/ physician assistants	◯	◯	◯
Otolaryngology-specific nursing	◯	◯	◯

Primary healthcare professionals with specific otolaryngology training	○	○	○

What care do otolaryngology/ ear nose and throat physicians provide? (check all that apply)	☐ Clinic procedures<br>☐ Medical management<br>☐ Surgical management<br>☐ Audiology<br>☐ Vestibular diagnosis/ management<br>☐ Hearing aid fitting<br>☐ Cochlear implant management<br>☐ Telehealth/ virtual care
What care do audiologist service providers provide? (check all that apply)	☐ Universal newborn hearing screening<br>☐ Pediatric hearing screening<br>☐ Diagnostic audiology<br>☐ Hearing aid fitting<br>☐ Cochlear implant management<br>☐ Vestibular diagnosis/ management<br>☐ Telehealth/ virtual care
What care do speech language pathologists or speech therapists provide? (check all that apply)	☐ Audiology<br>☐ Speech rehabilitation<br>☐ Swallow rehabilitation<br>☐ Voice rehabilitation<br>☐ Treatment of language impairment<br>☐ Telehealth/ virtual care
What care do otolaryngology clinical officers/ physician assistants provide? (check all that apply)	☐ Clinic procedures<br>☐ Medical management<br>☐ Surgical management<br>☐ Audiology<br>☐ Vestibular diagnosis/ management<br>☐ Hearing aid fitting<br>☐ Cochlear implant management<br>☐ Telehealth/ virtual care
What care do otolaryngology-specific nursing provide? (check all that apply)	☐ Clinic procedures<br>☐ Medical management<br>☐ Surgical management<br>☐ Audiology<br>☐ Vestibular diagnosis/ management<br>☐ Hearing aid fitting<br>☐ Cochlear implant management<br>☐ Telehealth/ virtual care
What care do primary healthcare professionals with specific otolaryngology training provide? (check all that apply)	☐ Hearing screening<br>☐ Basic treatment of ear conditions<br>☐ Telehealth/ virtual care

Workforce Estimates

Can you estimate the number of practicing otolaryngology/ ear nose and throat physicians in your country?	◯ Yes<br>◯ No
What is the estimated number of otolaryngology/ ear nose and throat physicians in your country?	__________________________
What is the estimated percentage of otolaryngology/ ear nose and throat physicians who work the majority of their time in big cities/towns at a tertiary medical center?	0 50 100<br>*(Place a mark on the scale above)*
What is the estimated percentage of otolaryngology/ ear nose and throat physicians who work full or part time in the public sector?	0 50 100<br>*(Place a mark on the scale above)*
What percentage of otolaryngology/ ear nose and throat physicians are female?	0 50 100<br>*(Place a mark on the scale above)*
What percentage of otolaryngology/ ear nose and throat physicians were trained mostly outside your country?	0 50 100<br>*(Place a mark on the scale above)*
Which of the following best describes the source of these data for otolaryngology workforce estimates?	◯ Official government agency (e.g. Ministry of Health)<br>◯ Professional otolaryngology/ ear nose and throat (ENT) society or association<br>◯ Medical licensing board<br>◯ Other
Please describe the other source.	__________________________
Can you estimate the number of practicing audiologist service providers in your country?	◯ Yes<br>◯ No
What is the estimated number of audiologist service providers in your country?	__________________________
What is the estimated percentage of audiologist service providers who work the majority of their time in big cities/towns at a tertiary medical center?	0 50 100<br>*(Place a mark on the scale above)*
What is the estimated percentage of audiologist service providers who work full or part time in the public sector?	0 50 100<br>*(Place a mark on the scale above)*

What percentage of audiologist service providers are female?	0 50 100 (Place a mark on the scale above)
What percentage of audiologist service providers were trained mostly outside your country?	0 50 100 (Place a mark on the scale above)
Which of the following best describes the source of these data for audiologist service providers workforce estimates.	◯ Official government agency (e.g. Ministry of Health)<br>◯ Professional otolaryngology/ ear nose and throat (ENT) society or association<br>◯ Medical licensing board<br>◯ Other
Please describe the other source.	__________________________________
Can you estimate the number of practicing speech language pathologists in your country?	◯ Yes<br>◯ No
What is the estimated number of speech language pathologists/speech therapists in your country?	__________________________________
What is the estimated percentage of speech language pathologists/speech therapists who work the majority of their time in big cities/towns at a tertiary medical center?	0 50 100 (Place a mark on the scale above)
What is the estimated percentage of speech language pathologists/speech therapists who work full or part time in the public sector?	0 50 100 (Place a mark on the scale above)
What percentage of speech language pathologists/speech therapists are female?	0 50 100 (Place a mark on the scale above)
What percentage of speech language pathologists/speech therapists were trained mostly outside your country?	0 50 100 (Place a mark on the scale above)
Which of the following best describes the source of these data for the speech language pathologist workforce estimates.	◯ Official government agency (e.g. Ministry of Health)<br>◯ Professional otolaryngology/ ear nose and throat (ENT) society or association<br>◯ Medical licensing board<br>◯ Other
Please describe the other source.	__________________________________

Training Institutions

How many programs provide OHNS training in your country?

Includes either a single institution or region if a training program spans across multiple institutions.

____

What is the average number of graduates from OHNS training programs (surgeons, MMED, post-graduates) per year in your country?

____

What is the total number of hospitals that provide surgical care in your country, if known?

____

What is the total number of hospitals providing OHNS surgical care, if known?

____

Contact Information

Please provide your name.

____

Please provide your email address.

____
