# Supplementary material for: The Global Otolaryngology–Head and Neck Surgery Workforce
Source: JAMA Otolaryngol Head Neck Surg. 2023 Aug 31;149(10):904–11. doi: 10.1001/jamaoto.2023.2339 (PMC10472262; doi:10.1001/jamaoto.2023.2339)
Supplement: Supplement 2. — eFigure 1. Survey Responses by World Health Organization Region eFigure 2. Disease Management by Specialties Other Than Otolaryngology–Head and Neck Surgery eTable 1. Definitions of Care Providers eTable 2. Number of Otolaryngology–Head and Neck Surgery Providers and Provider Density by Country eTable 3. Duplicate Workforce Estimates and Sources eTable 4. Provider or Practice Characteristics eTable 5. Disease Coverage by Income Group [file jamaotolaryngolheadnecksurg-e232339-s002.pdf]

Supplementary Online Content

Petrucci B, Okerosi S, Patterson RH, et al. The global otolaryngology–head and neck surgery workforce. *JAMA Otolaryngol Head Neck Surg*. Published online August 31, 2023. doi:10.1001/jamaoto.2023.2339

eFigure 1. Survey Responses by World Health Organization Region

eFigure 2. Disease Management by Specialties Other Than Otolaryngology–Head and Neck Surgery

eTable 1. Definitions of Care Providers

eTable 2. Number of Otolaryngology–Head and Neck Surgery Providers and Provider Density by Country

eTable 3. Duplicate Workforce Estimates and Sources

eTable 4. Provider or Practice Characteristics

eTable 5. Disease Coverage by Income Group

This supplementary material has been provided by the authors to give readers additional information about their work.

© 2023 Petrucci B et al. *JAMA Otolaryngology-Head & Neck Surgery*.

eFigure 1: Survey Responses by World Health Organization Region (%, Number of Countries with Responses in the Region)

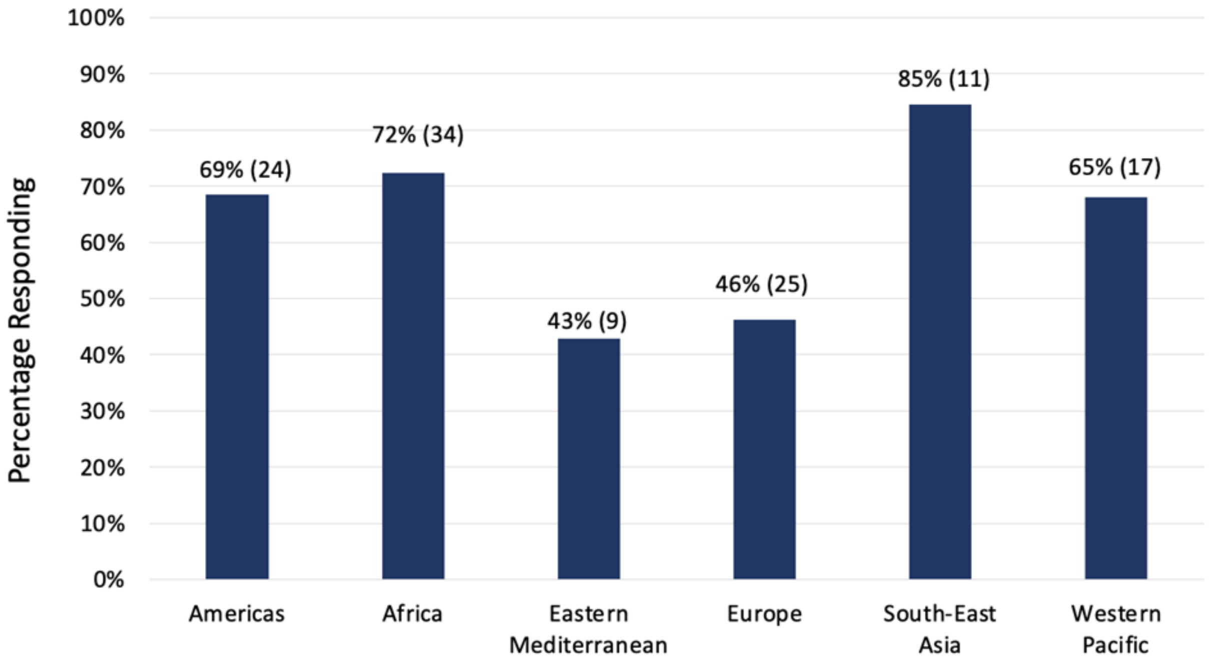

© 2023 Petrucci B et al. *JAMA Otolaryngology-Head & Neck Surgery*.

eFigure 2: Disease Management by Specialties Other Than Otolaryngology-Head and Neck Surgery

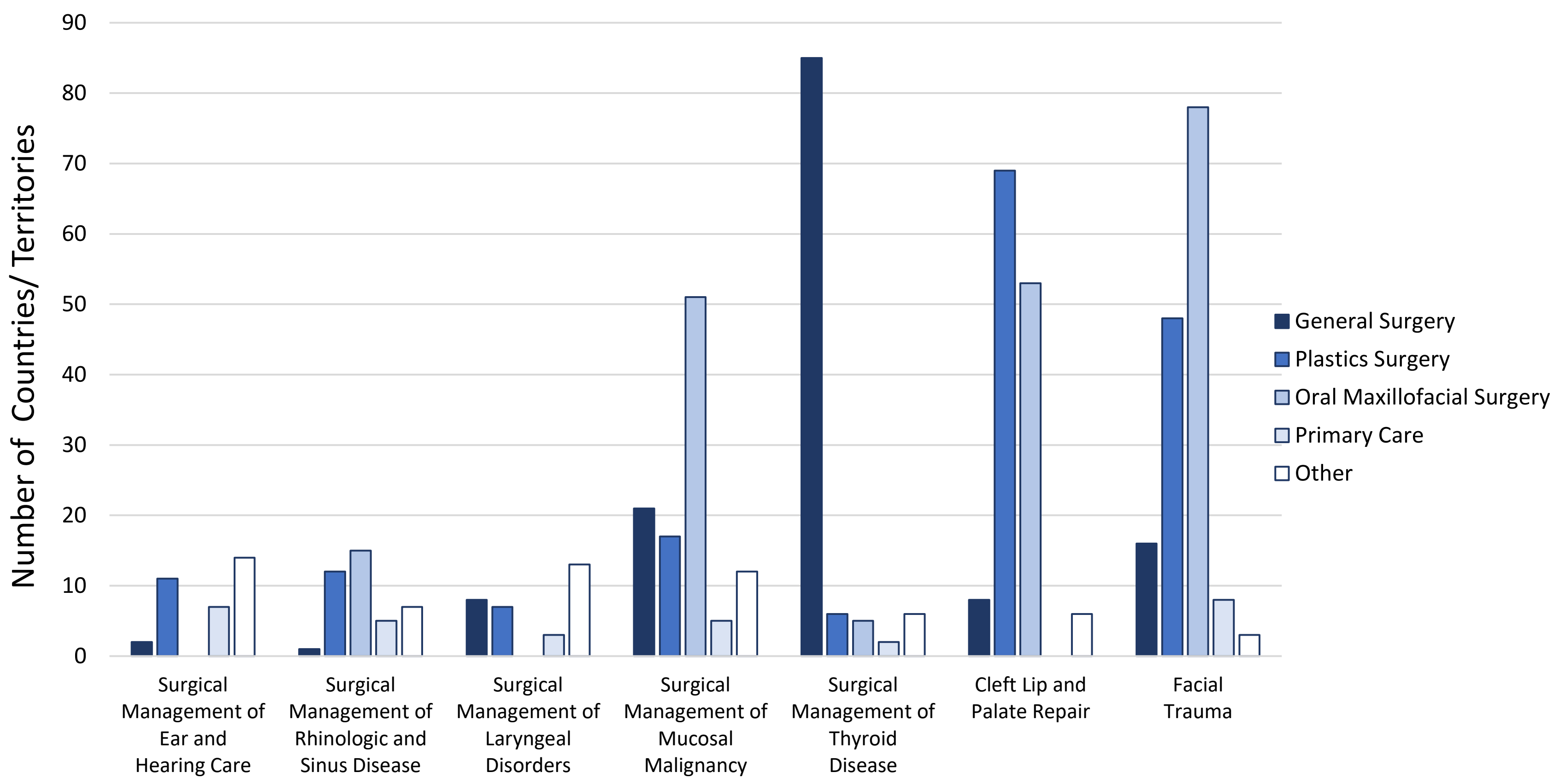

If respondents indicated that a disease process is not managed solely by otolaryngology-head and neck surgeons in their country/ territory, the respondant could select other surgical subspecialties that manage the disease processes.

© 2023 Petrucci B et al. *JAMA Otolaryngology-Head & Neck Surgery*.

eTable 1: Definitions of Care Providers

Item	Definition
Otolaryngology-head and neck surgery (OHNS)/ear nose and throat (ENT) physician	Doctor with a medical degree who has undergone specialized or accredited training in managing conditions of the ear, nose, and throat and head and neck. This does not include trainees.
Audiologist service provider	Allied health professional who has received specialized training in hearing assessment. This includes both audiologists and audiology technicians.
Speech language pathologists/speech therapists	Allied health professionals specializing in management of voice and swallowing
Otolaryngology clinical officers/ physician assistants	Allied health professionals who provide support of patients with head and neck conditions and who practice under the supervision of an OHNS/ENT
Otolaryngology-specific nursing	Nurses who have developed specialized skills in ear, nose, and throat care and/or spend the majority of their time with patients with head and neck conditions
Primary healthcare professionals with specific otolaryngology training	Primary care professionals who have undergone additional ear, nose, and throat training, which can include short courses.

© 2023 Petrucci B et al. *JAMA Otolaryngology-Head & Neck Surgery*.

eTable 2: Number of Otolaryngology-Head and Neck Surgery Providers and Provider Density by Country

World Health Organization Region	Country	Number of OHNS/ ENT Providers	Density of OHNS/ ENT per 100K Population	Population	World Bank Income Group
Americas	Argentina	1500	3.31	4.54E+07	upper middle income
Americas	Belize	5	1.23	4.05E+05	upper middle income
Americas	Bolivia	120	1.01	1.18E+07	lower middle income
Americas	Brazil	8000	3.76	2.13E+08	upper middle income
Americas	Canada	750	1.97	3.80E+07	high income
Americas	Chile	600	3.14	1.91E+07	high income
Americas	Colombia	850	1.67	5.09E+07	upper middle income
Americas	Costa Rica	110	2.16	5.09E+06	upper middle income
Americas	Cuba	628	5.54	1.13E+07	upper middle income
Americas	Dominican Republic	170	1.57	1.08E+07	upper middle income
Americas	Ecuador	400	2.27	1.76E+07	upper middle income
Americas	El Salvador	90	1.39	6.49E+06	lower middle income
Americas	Guatemala	48	0.28	1.69E+07	upper middle income
Americas	Guyana	2	0.25	7.90E+05	upper middle income
Americas	Haiti	30	0.26	1.14E+07	lower middle income
Americas	Honduras	110	1.11	9.90E+06	lower middle income
Americas	Mexico	4000	3.10	1.29E+08	upper middle income
Americas	Nicaragua	80	1.21	6.63E+06	lower middle income
Americas	Panama	80	1.85	4315000	high income
Americas	Paraguay	126	1.77	7133000	upper middle income
Americas	Peru	200	0.61	3.30E+07	upper middle income
Americas	United States of America	11000	3.31	3.32E+08	high income
Americas	Uruguay	160	4.59	3.49E+06	high income
Americas	Venezuela	458	1.61	2.84E+07	
Africa	Algeria	250	0.56	4.46E+07	lower middle income
Africa	Angola	45	0.14	3.29E+07	lower middle income
Africa	Burundi	7	0.06	1.19E+07	low income
Africa	Cameroon	300	1.13	2.65E+07	lower middle income
Africa	Cape Verde	5	0.91	5.50E+05	lower middle income
Africa	Chad	5	0.03	1.64E+07	low income
Africa	Republic of the Congo	80	1.41	5.66E+06	lower middle income
Africa	Democratic Republic of the Congo	24	0.03	9.24E+07	low income
Africa	Côte d'Ivoire	70	0.27	2.64E+07	lower middle income
Africa	Eritrea	2	0.06	3.21E+06	low income
Africa	Ethiopia	63	0.05	1.15E+08	low income
Africa	Gambia	0	0.00	2.42E+06	low income
Africa	Ghana	32.5	0.10	3.11E+07	lower middle income
Africa	Guinea	12	0.09	1.31E+07	low income
Africa	Kenya	120	0.22	5.38E+07	lower middle income
Africa	Liberia	1	0.02	5.06E+06	low income

© 2023 Petrucci B et al. *JAMA Otolaryngology-Head & Neck Surgery*.

Africa	Madagascar	21.5	0.08	2.77E+07	low income
Africa	Malawi	5	0.03	1.91E+07	low income
Africa	Mali	39	0.19	2.03E+07	low income
Africa	Mauritius	20	1.58	1.27E+06	upper middle income
Africa	Mozambique	24	0.07	3.22E+07	low income
Africa	Namibia	15	0.59	2.54E+06	upper middle income
Africa	Nigeria	250	0.12	2.06E+08	lower middle income
Africa	Rwanda	22	0.17	1.30E+07	low income
Africa	Senegal			1.72E+07	lower middle income
Africa	Sierra Leone	2	0.03	7.98E+06	low income
Africa	South Africa	260	0.44	5.93E+07	upper middle income
Africa	South Sudan	5	0.04	1.12E+07	low income
Africa	Swaziland	3	0.26	1.16E+06	lower middle income
Africa	Tanzania	55	0.09	5.97E+07	lower middle income
Africa	Togo	16	0.19	8.48E+06	low income
Africa	Uganda	45	0.10	4.57E+07	low income
Africa	Zambia	6	0.03	1.84E+07	low income
Africa	Zimbabwe	16.3	0.11	1.49E+07	lower middle income
Eastern Mediterranean	Egypt	3000	2.93	1.02E+08	lower middle income
Eastern Mediterranean	Iraq			4.12E+07	upper middle income
Eastern Mediterranean	Kuwait	300	6.93	4.33E+06	high income
Eastern Mediterranean	Lebanon	180	2.66	6.77E+06	lower middle income
Eastern Mediterranean	Libya			6959000	upper middle income
Eastern Mediterranean	Qatar			2931000	high income
Eastern Mediterranean	Saudia Arabia			3.53E+07	high income
Eastern Mediterranean	Sudan	160	0.36	4.38E+07	low income
Eastern Mediterranean	Syrian Arab Republic (Syria)			1.83E+07	low income
Europe	Albania	94	3.31	2.84E+06	upper middle income
Europe	Bulgaria	250	3.61	6934000	upper middle income
Europe	Croatia	240	6.16	3899000	high income
Europe	Cyprus	70.5	5.84	1207000	high income
Europe	Czech Republic	825	7.71	1.07E+07	high income
Europe	Denmark	600	10.29	5.83E+06	high income
Europe	Estonia	100	7.52	1329000	high income
Europe	Finland	350	6.33	5529000	high income
Europe	France	5000	7.41	6.75E+07	high income
Europe	Georgia	200	5.39	3.71E+06	upper middle income
Europe	Germany	7500	9.01	8.32E+07	high income
Europe	Greece			1.07E+07	high income
Europe	Ireland	60	1.19	5.03E+06	high income
Europe	Israel	500	5.42	9217000	high income
Europe	Italy	3000	5.08	5.91E+07	high income
Europe	Kazakhstan	1200	6.40	1.88E+07	upper middle income
Europe	Malta	20	3.88	5.15E+05	high income
Europe	Norway	500	9.30	5379000	high income
Europe	Romania	800	4.15	1.93E+07	high income
Europe	Slovakia	500	9.16	5.46E+06	high income

© 2023 Petrucci B et al. *JAMA Otolaryngology-Head & Neck Surgery*.

Europe	Slovenia	100	4.76	2100000	high income
Europe	Spain	3000	6.34	4.74E+07	high income
Europe	Sweden	700	6.76	1.04E+07	high income
Europe	Turkey	3200	3.79	8.43E+07	upper middle income
Europe	United Kingdom	1200	1.78	6.73E+07	high income
Southeast Asia	Bangladesh	600	0.36	1.65E+08	lower middle income
Southeast Asia	Bhutan	7	0.90	7.80E+05	lower middle income
Southeast Asia	India	15000	1.09	1.38E+09	lower middle income
Southeast Asia	Indonesia	1600	0.58	2.74E+08	lower middle income
Southeast Asia	Korea (South, Republic of Korea)	3000	5.79	5.18E+07	high income
Southeast Asia	Maldives	15	2.77	5.41E+05	upper middle income
Southeast Asia	Myanmar (Burma)	200	0.37	5.44E+07	lower middle income
Southeast Asia	Nepal	255	0.88	2.91E+07	lower middle income
Southeast Asia	Sri Lanka	60	0.27	2.22E+07	lower middle income
Southeast Asia	Thailand	2000	2.87	6.98E+07	upper middle income
Southeast Asia	Hong Kong	180	2.41	7.48E+06	high income
Western Pacific	Australia	600	2.33	2.57E+07	high income
Western Pacific	Cambodia	70	0.41	1.69E+07	lower middle income
Western Pacific	China	40000	2.84	1.41E+09	upper middle income
Western Pacific	Fiji	1	0.11	8.96E+05	upper middle income
Western Pacific	Japan	11000	8.74	1.26E+08	high income
Western Pacific	Kiribati	0	0.00	1.19E+05	lower middle income
Western Pacific	Malaysia	450	1.39	3.24E+07	upper middle income
Western Pacific	Marshall Islands	1	1.69	5.90E+04	upper middle income
Western Pacific	New Zealand	125	2.46	5084000	high income
Western Pacific	Niue	1	61.73	1620	
Western Pacific	Papua New Guinea	7	0.08	9119000	lower middle income
Western Pacific	Philippines	890	0.80	1.11E+08	lower middle income
Western Pacific	Samoa	1	0.51	1.98E+05	lower middle income
Western Pacific	Singapore	180	3.17	5686000	high income
Western Pacific	Solomon Islands	1	0.15	687000	lower middle income
Western Pacific	Tonga	2	1.89	106000	upper middle income
Western Pacific	Vietnam	1800	1.83	9.82E+07	lower middle income
	Taiwan	2650	11.24	2.36E+07	

© 2023 Petrucci B et al. *JAMA Otolaryngology-Head & Neck Surgery*.

eTable 3: Duplicate Workforce Estimates and Sources

Country	Workforce Estimate	Source
Congo (Democratic Republic of the Congo)	15, 27, 30	Official government agency (e.g. Ministry of Health)
Cyprus	70, 71	Professional otolaryngology/ ear nose and throat society or association
Czech Republic	650, 1000	Professional otolaryngology/ ear nose and throat society or association
Ghana	20, 45	Professional otolaryngology/ ear nose and throat society or association
Madagascar	20, 21	Professional otolaryngology/ ear nose and throat society or association
Malaysia	400, 500	Professional otolaryngology/ ear nose and throat society or association
Myanmar (Burma)	150, 250	Professional otolaryngology/ ear nose and throat society or association
New Zealand	120, 130	Professional otolaryngology/ ear nose and throat society or association
	160	Other
Panama	80	Professional otolaryngology/ ear nose and throat society or association
	60	Official government agency (e.g. Ministry of Health)
Sierra Leone	2, 2	Official government agency (e.g. Ministry of Health)
Spain	3000, 3000	Professional otolaryngology/ ear nose and throat society or association
	2200	Medical licensing board
	3000	Other
Taiwan	2300, 3000	Professional otolaryngology/ ear nose and throat society or association
Uganda	25, 45, 65	Professional otolaryngology/ ear nose and throat society or association
Vietnam	1800	Professional otolaryngology/ ear nose and throat society or association
	1500	Official government agency (e.g. Ministry of Health)
Zambia	6	Professional otolaryngology/ ear nose and throat society or association
	6	Medical licensing board
Zimbabwe	15, 16, 18	Professional otolaryngology/ ear nose and throat society or association
	15	Medical licensing board

© 2023 Petrucci B et al. *JAMA Otolaryngology-Head & Neck Surgery*.

eTable 4: Provider or Practice Characteristics

**Overall**	**Number of Countries/ Territories***	**Mean (%)**	**Median (%)**	**Interquartile Range (%)**	**Range Among Countries/ Territories (%)**
Female	99	39.8	40.0	30.0 – 42.0	0-100
Urban (providers who work the majority of their time in big cities/towns at a tertiary medical center)	97	50.2	50.0	46.0 – 65.0	14-100
Public Sector (providers who work full or part time in the public sector)	97	56.5	70.0	30.0 – 80.0	0-100
Proportion of Workforce Trained Outside of the Country (providers who were otolaryngology-specialty trained outside your country)	99	17.3	5.0	5.0 – 30.0	0-100

*One response per country/ territory was used for these estimates.

© 2023 Petrucci B et al. *JAMA Otolaryngology-Head & Neck Surgery*.

eTable 5: Disease Coverage by Income Group

	Proportion of Diseases Managed by OHNS Providers in the Country/ Territory	World Bank Income Group				Cliff's Delta Effect Size and 95% Confidence Interval*
		High	Upper-middle	Lower-middle	Low	
Surgical Management of Ear and Hearing Care Diseases	None	0 (0%)	1 (3%)	0 (0%)	2 (11%)	**0.24 (0.12,0.35)**
	Less than/about 50%	1 (3%)	6 (20%)	7 (22%)	6 (32%)	
	Over 50%/ All	31 (97%)	23 (77%)	25 (78%)	11 (58%)	
Surgical Management of Rhinologic and Sinus Disease	None	0 (0%)	0 (0%)	0 (0%)	1 (5%)	**0.17 (0.06,0.27)**
	Less than/about 50%	1 (3%)	4 (13%)	6 (19%)	5 (26%)	
	Over 50%/ All	31 (97%)	26 (87%)	26 (81%)	13 (68%)	
Surgical Management of Benign Laryngeal Disorders	None	0 (0%)	1 (3%)	2 (6%)	2 (11%)	**0.22 (0.08,0.36)**
	Less than/about 50%	3 (9%)	7 (23%)	6 (18%)	8 (42%)	
	Over 50%/ All	29 (91%)	22 (73%)	25 (76%)	9 (47%)	
Surgical Management of Upper Aerodigestive Malignancies	None	0 (0%)	2 (7%)	0 (0%)	2 (11%)	**0.24 (0.07,0.39)**
	Less than/about 50%	4 (12%)	5 (17%)	13 (41%)	7 (37%)	
	Over 50%/ All	28 (88%)	22 (76%)	19 (59%)	10 (53%)	
Surgical Management of Thyroid Diseases	None	2 (6%)	3 (10%)	2 (6%)	1 (6%)	-0.03 (-0.23,0.17)
	Less than/about 50%	18 (56%)	18 (60%)	17 (52%)	7 (39%)	
	Over 50%/ All	12 (38%)	9 (30%)	14 (42%)	10 (56%)	
Cleft Lip and Palate Repair	None	9 (28%)	13 (43%)	8 (25%)	2 (11%)	-0.13 (-0.27,0.03)
	Less than/about 50%	19 (59%)	12 (40%)	15 (47%)	10 (56%)	
	Over 50%/ All	4 (12%)	5 (17%)	9 (28%)	6 (33%)	
Facial Trauma	None	0 (0%)	1 (3%)	1 (3%)	1 (6%)	-0.09 (-0.25,0.08)
	Less than/about 50%	26 (81%)	22 (76%)	20 (62%)	12 (67%)	
	Over 50%/ All	6 (19%)	6 (21%)	11 (34%)	5 (28%)	

* The data was dichotomized for the Cliff's Delta effect size calculation. Proportion of OHNS coverage was dichotomized to >50% and ≤50% OHNS coverage. Country income was dichotomized to high-income countries and the remaining were groups into low-and

© 2023 Petrucci B et al. *JAMA Otolaryngology-Head & Neck Surgery*.

middle-income countries. Values greater than 0 indicate that the high-income countries had more OHNS providers managing the disease. Values less than 0 indicate low and middle-income countries had more OHNS provides managing the disease.

© 2023 Petrucci B et al. *JAMA Otolaryngology-Head & Neck Surgery*.
