# Supplementary material for: The Global Otolaryngology–Head and Neck Surgery Workforce
Source: JAMA Otolaryngol Head Neck Surg. 2023 Aug 31;149(10):904–11. doi: 10.1001/jamaoto.2023.2339 (PMC10472262; doi:10.1001/jamaoto.2023.2339)
Supplement: Supplement 3. — Data Sharing Statement [file jamaotolaryngolheadnecksurg-e232339-s003.pdf]

Data Sharing Statement

Petrucci. The Global Otolaryngology–Head and Neck Surgery Workforce. *JAMA Otolaryngol Head Neck Surg*. Published August 31, 2023. doi:10.1001/jamaoto.2023.2339

Data
Data available: Yes
Data types: Deidentified participant data, Data dictionary
How to access data: maryjue.xu@ucsf.edu
When available: With publication

Supporting Documents
Document types: Statistical/analytic code
How to access documents: maryjue.xu@ucsf.edu
When available: With publication

Additional Information
Who can access the data: Researchers, policy makers, and clinicians whose proposed use of the data has been approved.
Types of analyses: For purposes of policy, advocacy, and research.
Mechanisms of data availability: With investigator support, after approval of a proposal.
